# Peritoneal Dialysis‐Related *Mycobacterium fortuitum* Exit‐Site/Tunnel Infection in a Pediatric Patient: A Case Report

**DOI:** 10.1002/ccr3.71975

**Published:** 2026-02-12

**Authors:** Rina Takahashi, Hiroshi Tamura, Keishiro Furuie, Hiroko Nagata, Shohei Kuraoka

**Affiliations:** ^1^ Department of Pediatrics, Faculty of Life Sciences Kumamoto University Kumamoto Japan

**Keywords:** case report, children, nontuberculous *Mycobacteria* infection, peritoneal dialysis

## Abstract

In patients in peritoneal dialysis (PD) who develop exit site and/or catheter tunnel infections, expeditious identification of the causative organism and early implementation of treatment are important to ensure good outcomes. Among the many causative organisms, the diagnosis of *Mycobacterium fortuitum* is particularly challenging, with its treatment often requiring a combination of approaches, including the removal of the peritoneal catheter and the administration of multiple antibiotics. Here, we present the case of a 25‐month‐old pediatric patient on PD who developed PD‐associated tunnel infection due to *M. fortuitum* ; the patient was successfully treated with multimodal antibiotics, with no sequela, following the rapid identification of the causative agent, the use of multimodal antibiotics, and PD catheter removal. In our case, the timely initiation of treatment, combined with ultrasound evaluation, was crucial for achieving a favorable outcome.

## Key Clinical Message

Culture‐negative ESI/TI that does not respond to standard antibiotics should be suspected for NTM infection. In patients with NTM infection, we suggest catheter removal and a multi‐combination antibiotic regimen. In our case, the timely initiation of treatment, combined with ultrasound evaluation, was crucial for achieving a favorable outcome.

**Abbreviations**
AMK: amikacin
AZM: azithromycin hydrate
CAKUT: congenital anomalies of the kidney and urinary tract
CAM: clarithromycin
CPFX: ciprofloxacin hydrochloride
CRP: C‐reaction protein
DM: diabetes mellitus
DOXY: doxycycline
EB: ethambutol
ESI: exit‐site infection
GM: gentamicin
GN: glomerulonephritis
HD: hemodialysis
IPM/CS: imipenem/cilastatin sodium
KT: kidney transplantation
LVFX: levofloxacin hydrate
MEPM: meropenem hydrate
NTM: nontuberculous *Mycobacteria*
PD: peritoneal dialysis
REP: rifampicin
RGM: rapid‐growing nontuberculous *Mycobacteria*
SGM: slow‐growing nontuberculous *Mycobacteria*
TGC: tigecycline
TI: tunnel infection

## 1 Introduction

Patients on peritoneal dialysis (PD), a widely used renal replacement therapy worldwide, can develop a range of infections, including peritonitis, tunnel infection (TI), and exit site infection (ESI). ESIs and TIs are strongly associated with the development of peritonitis in patients with PD, often leading to the impairment of PD ultrafiltration and solute clearance, increased hospitalization, and high mortality [1, 2]. Therefore, the identification of causative organisms and the early administration of antibiotics are essential to minimize the risk of treatment failure [3, 4].

*Mycobacterium fortuitum* ( *M. fortuitum* ) refers to a group of nontuberculous mycobacteria (NTM) that are found in water, soil, and aerosols, which are also some of the most drug‐resistant microorganisms [5]. NTM account for approximately 3% of all cases of PD‐associated peritonitis [6], *M. fortuitum* has become one of the most common causes of PD‐associated peritonitis [7, 8], and the diagnosis of bacterial peritonitis caused by NTM is challenging.

The eradication of NTM infections in patients on PD is particularly difficult and often requires the removal of the peritoneal catheter and the administration of multiple antibiotics. Established treatment regimens for NTM infections are lacking in patients with PD, and the clinical course of NTM‐associated infections remains unclear.

Here, we report the case of a 25‐month‐old pediatric patient on PD for end‐stage kidney failure due to steroid‐resistant nephrotic syndrome who developed PD‐associated NTM infection, which was successfully treated with multimodal antibiotics.

## 2 Case Presentation/Examination

The patient was a 25‐month‐old boy with a height of 79.5 cm (−2.2 SD) and a weight of 10.8 kg (−0.8 SD), who was diagnosed with nephrotic syndrome at 17 months of age. He was subsequently referred to our hospital for steroid‐resistant nephrotic syndrome. After detailed examination, he did not respond to steroid treatment and underwent renal biopsy, which revealed focal segmental glomerulosclerosis. He was initiated on continuous hemodialysis at 21 months of age due to renal failure and overflow. Following the insertion of a double‐cuff straight Tenckhoff catheter, he was initiated on PD at 22 months of age. Drug therapy with albumin and gamma globulin supplementation was continued alongside PD.

**FIGURE 1:**
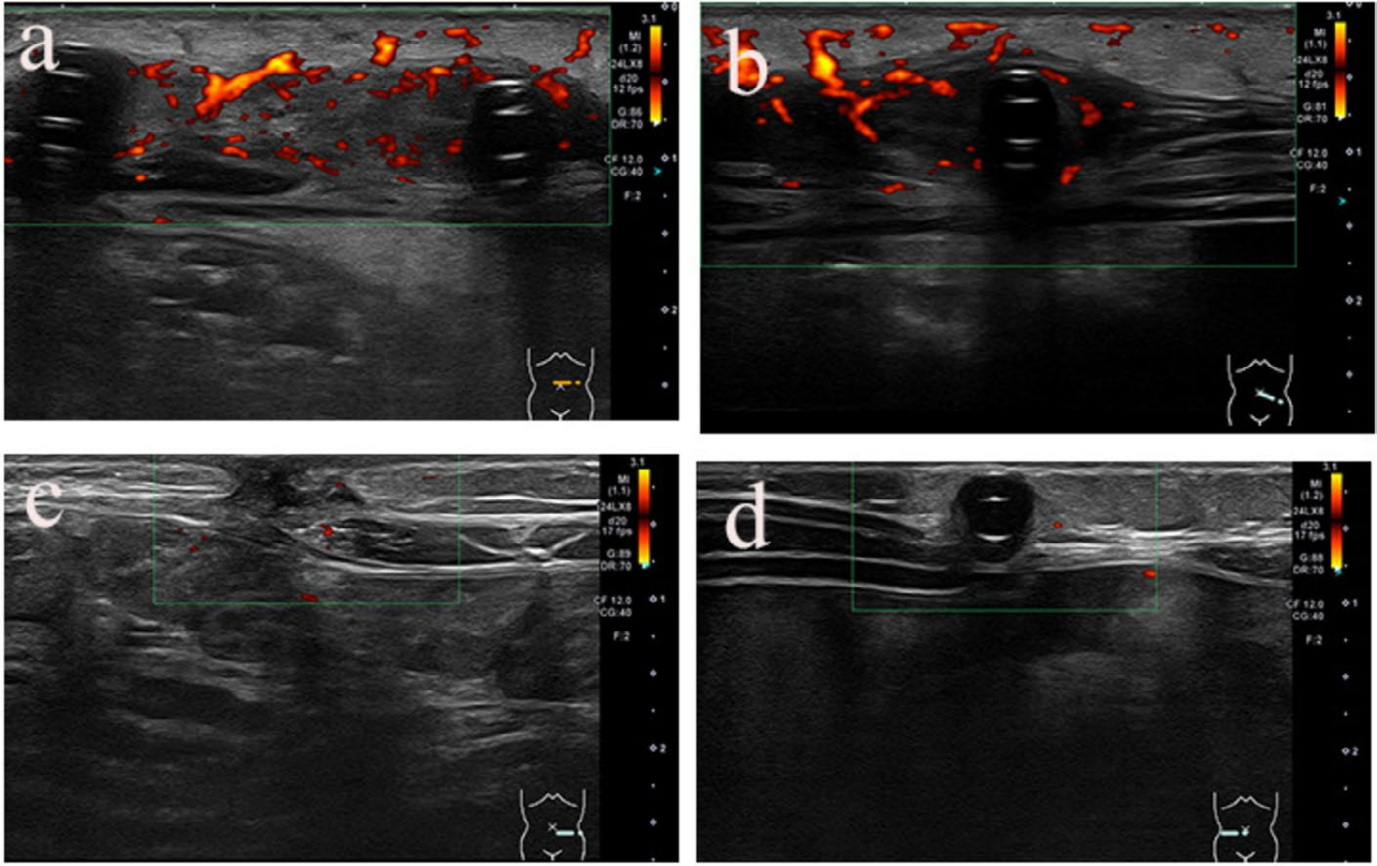
(a, b) A hypoechoic area was observed around the cuff, and blood flow signals were observed in the same area. (c) There was a hypoechoic area where the cuff had been located, and a slight blood flow signal was observed. (d) No signs of infection were found in the new catheter area.

At 25 months of age, the patient underwent unilateral nephrectomy to treat hypoalbuminemia. Although the postoperative course was uneventful, purulent discharge from the PD catheter exit site was noted approximately 1 week after surgery. The patient did not have fever, abdominal pain, or cloudy drainage fluid. After collecting samples for bacterial cultures, treatment with gentamycin was initiated to treat the PD catheter ESI. However, ultrasound examination, which was performed 6 days later due to the lack of improvement, revealed a subcutaneous infection (Figure 1a); therefore, intraperitoneal vancomycin (VCM) (30 mg/kg) administration was initiated.

## 3 Methods (Differential Diagnosis, Investigations and Treatment)

The bacterial culture results obtained 10 days after the initiation of gentamicin treatment revealed *M. fortuitum* as the causative agent. VCM and gentamicin were discontinued, and amikacin (AMK) (5 mg/kg/day) infusion and oral norfloxacin (NFLX) were initiated for NTM treatment. However, no improvement was observed in the exit site 3 days after the change in treatment; therefore, NFLX was replaced with ciprofloxacin (CPFX) (10 mg/kg/day) infusion and oral clarithromycin (CAM) was added. Ultrasound examination on the 12th day of NTM treatment revealed a TI (Figure 1b), and the PD catheter was removed on the 14th day of NTM treatment. Additionally, a long‐term indwelling blood access catheter was inserted and the patient was placed on hemodialysis (HD). No change to the antibiotic regimen was necessary based on the susceptibility test results for *M. fortuitum* , which were obtained 2 weeks after the NTM treatment initiation (Table 1).

**TABLE 1:** Susceptibility testing results for *Mycobacterium fortuitum* isolate.

Drug	MIC (mg/mL)
Amikacin	≤ 4
Tobramycin	4
Imipenem	≤ 2
Levofloxacin	≤ 1
Moxifloxacin	≤ 0.25
Sulfamethoxazole/trimethoprim	> 160
Doxycycline	< 0.5
Meropenem	8
Linezolid	8
Clofazimine	0.5
Sitafloxacin	≤ 0.25
Azithromycin	2
Clarithromycin	0.5

Abbreviation: MIC, Minimum inhibitory concentration.

Due to the minimal improvement in drainage from the exit site, subcutaneous debridement was performed under general anesthesia on the 20th day of NTM treatment and CAM was switched to intravenous imipenem/cilastatin (IPM/CS) (20 mg/kg/day). Ultrasound examination performed 60 days after the PD catheter removal confirmed the absence of TI (Figure 1c), and the PD catheter was reinserted. Two weeks later, the patient was switched from HD to PD.

## 4 Conclusion and Results (Outcome and Follow‐Up)

**FIGURE 2:**
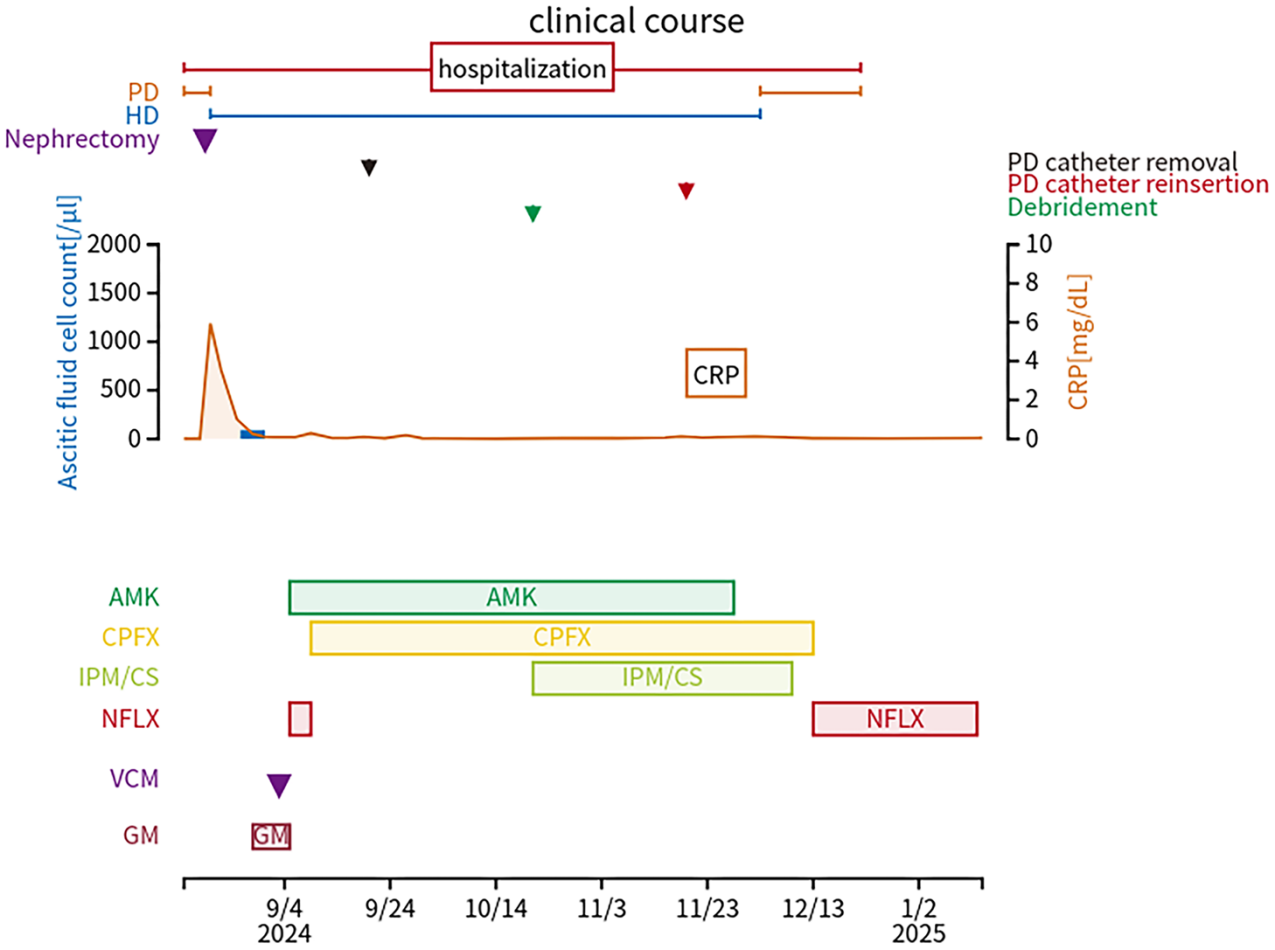
Clinical course.

Thereafter, the patient's clinical progress was good, and AMK was used for 12 weeks before discontinuation. One week later, IPM/CS was discontinued, CPFX was changed to oral NFLX, and the patient was discharged. Thereafter, the patient was followed on an outpatient basis, confirming the absence of TI (Figure 1d) with negative exit‐site and ascites cultures; therefore, NFLX was discontinued after 4 weeks of treatment. The patient has not experienced recurrence since then. Figure 2 shows the clinical course of the patient.

## 5 Discussion

*M. fortuitum* are rapidly growing mycobacteria (RGM), which can be isolated from soil, dust, water, terrestrial and aquatic animals, hospital environments, and contaminated reagents and medicines [9, 10]. In patients on long‐term dialysis, most of the reported cases of NTM infections are associated with ESI and peritonitis [11, 12, 13, 14, 15, 16, 17, 18, 19, 20] although other rare infections, such as skin infections [13, 14], septic arthritis [15], synovitis, and spondylitis [16], have also been reported. *M. fortuitum* , *M. abscessus* , and *M. chelonae* are the most commonly encountered NTM in clinical practice, whereas slow‐growing mycobacteria (SGM), such as *M. avium* complex, *M. gordonae* , and *M. kansasii* , have also been reported [15, 16, 17, 18, 19, 20]. RGM can be detected by Gram staining and are mistaken for diphtheroid in some cases. However, RGM also form visible colonies on regular culture media, such as blood and chocolate agar, after approximately 1 week of culture. Ziehl–Neelsen staining is useful in differentiating *M. tuberculosis* from other bacteria, whereas Ziehl–Neelsen staining and *M. tuberculosis* culture are essential for the identification of SGM. Therefore, in the presence of clinical suspicion for ESI caused by NTM, cultures should be performed on routine bacterial culture media for an extended period, and culture for *M. tuberculosis* should be requested.

A comprehensive search was conducted using PubMed and Google Scholar databases, covering publications up to December 31, 2024. The search terms included “dialysis,” “peritoneal dialysis,” and “mycobacterium.” The comprehensive reviews of existing literature conducted by these authors confirmed that PD catheter removal was defined as the extraction of the PD catheter following the diagnosis of NTM peritonitis during an active peritonitis episode. NTM peritonitis‐related mortality was reported as death occurring during the treatment period, from the time of infection onset or diagnosis. Table 2 summarizes a total of 49 reported cases fulfilling the search criteria, including the self‐examination cases (Figure S1, Data S1). Briefly, 65% of the patients were male, with an average age of 56 years at onset. The causes of renal failure were diabetes mellitus and glomerulonephritis in 36% and 19% of the cases, respectively. The average treatment duration was 3 months, and 98% of the cases were cured. The main causes of NTM‐associated PD infections were RGM, most commonly *M. fortuitum* , followed by *M. abscessus* and *M. chelonae* . Conversely, in a small number of cases, the causative agent was *M. avium* , an SGM that is a primary pathogen causing pulmonary NTM disease (Table 3). These differences in prevalence may reflect the varying ecological niches of different NTM species.

**TABLE 2:** Clinical characteristics of patients with PD‐related NTM ESI/TI.

	ESI/TI
Age (year)	Mean 56.24 ± 19.27
	Median 61 (0.9~89)
	*N* = 49
Sex	Male 32/49 (65%)
Kidney disease	DM 15/42 (36%)
	GN 8/42 (19%)
	Sclerosis 2/42 (5%)
	CAKUT 3/42 (7%)
	Lupus nephritis 4/42 (10%)
	Others 10/42 (24%)
Dialysis duration before onset of NTM (months)	Mean 31.59 ± 34.53
	Median 18 (1~156)
	*N* = 41
RGM/(RGM+SGM)	48/49 (98%)
Duration from disease onset to anti NTM treatment (days)	Mean 16.4 ± 13.69
	Median 12 (5~63)
	*N* = 21
Catheter remove	21/49 (43%) replacement 8/49 (16%)
Duration medication (months)	Mean 2.91 ± 2.30
	Median 2 (0~10.5)
	*N* = 40
Outcome	Recovery 48/49 (98%)
	HD 8/47 (17%)
	HD 10/38→PD 38/47 (81%)
	HD 1/1→KT 1/47 (2%)
	All‐cause mortality 1/49 (2%)^a^

*Note:* All‐cause mortality (death from NTM peritonitis or while receiving antituberculosis treatment for NTM peritonitis).

^a^ The three patients whose PD catheters were not removed did not consent to removal and died.

**TABLE 3:** Classification of causative bacteria in 49 cases of atypical mycobacterial ESI/TI.

		ESI/TI
SGM Photochromogens, Runyon group 1	*M. kansasii*	0
SGM Scotochromogens, Runyon group 2	*M. gordonae*	1
SGM Nonchromogens, Runyon group 3	MAC	0
RGM Runyon group 4	*M. fortuitum*	21
	*M. abscessus*	16
	*M. chelonae*	8
	*M. immunogenum*	1
	Others	2

The International Society of Peritoneal Dialysis (ISPD) guidelines recommend the daily application of antibacterial ointment to the exit site for patients with ESI [21], although some studies suggest that exit‐site care using gentamicin may lead to NTM infection [22, 23]. In the present case, the high dose of topical gentamicin might have led to selective pressure, promoting the growth of NTM. Indeed, our literature search identified one patient who spontaneously recovered from ESI/TI caused by NTM following the discontinuation of the topical gentamicin ointment, which might be due to the removal of selective pressure and due to the patient's autoimmune infection [24].

PD‐associated infections caused by NTM often hinder the ability to continue PD. The ISPD guidelines recommend the removal of the PD catheter in patients with NTM peritonitis [25] and a temporary switch to hemodialysis in patients with NTM ESI [26]. In this study, PD catheter removal and catheter replacement were performed in 43% and 16% of cases, respectively. The PD catheter was removed and HD was initiated in 19 patients, of whom 10 resumed PD and 1 underwent kidney transplantation. There were five children under the age of 15, including our case, and PD was resumed in all of them. In pediatric patients, following PD catheter removal, a temporary transition to HD followed by PD catheter reinsertion or kidney transplantation should be considered. However, HD may be challenging in pediatric patients and is often performed only in specialized facilities. Several studies reported the challenges in managing pediatric patients on HD, who were successfully treated while on PD. The main factors enabling PD continuation without the need to switch to hemodialysis include PD catheter replacement before the onset of peritonitis, debridement of the infected site, and long‐term administration of multiple antibiotics based on drug susceptibility testing [27].

It has been reported that ultrasound examination has enabled the establishment of appropriate treatments for cutaneous tuberculosis infection [28, 29]. Ultrasound examination of the catheter tunnel is a useful approach in patients on PD with catheter‐associated infections. In patients with ESI, ultrasound examination is a more sensitive method for the diagnosis of concomitant TI compared with the assessment based on clinical parameters alone. Additionally, ultrasound examination is important for a more accurate distinction between an ESI that is likely to be resolved with oral antibiotic therapy from one that may require more aggressive treatment, such as intravenous antibiotic therapy, surgical correction of the catheter tunnel, and catheter removal. In patients with TIs, ultrasound examination allows for the accurate localization of the infected catheter segment and guides the clinicians who are considering a surgical approach in patients for whom the ultrasound findings do not change over time. In such cases, repeat ultrasound examination can be performed 2 weeks after the start of antibiotic treatment to monitor treatment response and guide decisions regarding the extension of antibiotic therapy versus surgical intervention. Conversely, in patients with peritonitis, the evidence on the usefulness of the tunnel remains limited; however, the detection of tunnel lesions provides important prognostic information for clinicians to select the optimal treatment. However, there is no evidence to support the use of tunnel ultrasound as a screening tool for the early detection of TI in asymptomatic patients [30]. The utility of ultrasound in the diagnosis and management of peritoneal catheter‐associated infections is unquestionable, although rigorously designed studies in larger cohorts are necessary to confirm the available evidence.

There is no consensus on the optimal antibiotic regimens, combination therapies, and treatment duration for PD‐associated NTM infections [31]. The most commonly used antibiotics are AMK, imipenem, cefoxitin, and macrolides for RGM infections (Table 4). Although most infections are susceptible to these drugs, the susceptibility patterns vary depending on the site of infection and other factors. Therefore, multidrug therapy based on the results of susceptibility testing is the mainstay of treatment [29, 32]. The duration of antibiotic treatment varies depending on the site of infection, and long‐term administration is required for at least several months in most cases [7, 31, 33, 34]. AMK is a key drug in RGM infections and may require long‐term administration. Particularly in children, attention should be paid to amikacin‐induced nephrotoxicity and blood drug concentrations should be monitored in patients treated with AMK. Further studies are warranted to elucidate the appropriate antibiotic therapy and the proper administration duration, with consideration of the treatment side effects.

**TABLE 4:** Literature review of treatment of NTM ESI/TI.

	Aminoglycoside	Macrolides		New quinolone		Carbapenem		Tetracycline		Antituberculous drugs	
	AMK	CAM	AZM	CPFX	LVFX	IPM/CS	MEPM	DOXY	TGC	REP	EB
*M. fortuitum* 21	11	14	1	9	2	2	0	0	0	2	0
*M. abscessus* 16	9	12	3	1	1	5	4	1	2	1	1
*M. chelonae* 8	6	8	0	0	0	0	0	1	0	1	1

## 6 Conclusions

**FIGURE 3:**
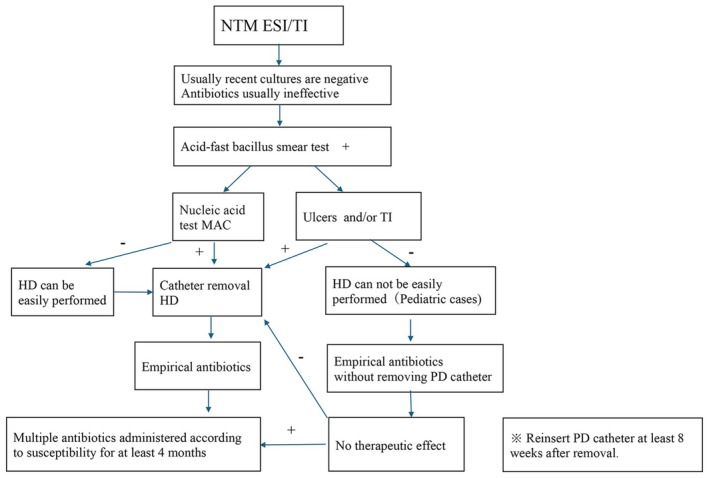
Management algorithm for NTM ESI/TI. (1) Suspect NTM infection when cultures are negative and treatment fails. (2) If the acid‐fast staining is positive, use a PCR test to determine whether it is TB or Mac. (3) If Mac is positive, or there is an ulcer or TI (Tunnel infection has spread to the deep cuff), the PD catheter should be removed. (4) Begin Administering empirical antibiotics for NTM. If the PD catheter is still in place, it should be removed if the treatment is no longer effective after 2 weeks. (5) If sensitivity is determined, adjust the antibiotic accordingly. (6) Take multiple antibiotics for at least 4 months.

Culture‐negative peritonitis and ESI/TI that do not respond to standard antibiotic therapy should be evaluated for NTM infections. The ISPD guidelines recommend the removal of the PD catheter with a temporary switching to hemodialysis in patients with ESI/TI caused by NTM. However, implementing hemodialysis can be challenging in pediatric cases. In our case, the timely initiation of treatment, combined with ultrasound evaluation, was crucial for achieving a favorable outcome. In patients with suspicious TI caused by NTM, we suggest catheter removal and the initiation of a multimodal antibiotic regimen for at least 4 months (Figure 3).

## Author Contributions

**Rina Takahashi:** data curation, writing – original draft. **Hiroshi Tamura:** conceptualization, data curation, formal analysis, writing – review and editing. **Keishiro Furuie:** data curation. **Hiroko Nagata:** data curation. **Shohei Kuraoka:** data curation.

## Funding

The authors have nothing to report.

## Ethics Statement

All the procedures performed in studies involving human participants were conducted in accordance with the ethical standards of the Institutional Committee and the 1964 Declaration of Helsinki and its later amendments or comparable ethical standards (64th WMA General Assembly, Fortaleza, Brazil, October 2013).

## Consent

Written informed consent was obtained from his parents for the publication of this case report.

## Conflicts of Interest

The authors declare no conflicts of interest.

## Supporting information


**Data S1:** Supplementary references.

**Figure S1:** Literature search strategy illustrating the databases searched, search terms, and selection process used to identify eligible studies for this review.

## Data Availability

No data were used to support the findings of this study.
