# Supplementary material for: Peritoneal Dialysis‐Related Mycobacterium fortuitum Exit‐Site/Tunnel Infection in a Pediatric Patient: A Case Report
Source: Clin Case Rep. 2026 Feb 12;14(2):e71975. doi: 10.1002/ccr3.71975 (PMC12901669; doi:10.1002/ccr3.71975)
Supplement: Supplementary file 1 — Data S1: Supplementary references. [file CCR3-14-e71975-s001.docx]

**SUPPLEMENTARY REFERENCES S1**

1. Joseph P. Pulliam, M.D., Donald D. Vernon, M.D. et al. Nontuberculous mycobacterial peritonitis associated with continuous ambulatory peritoneal dialysis. Am J kid disease 1983 6; 610-614
2. I. M. LINTON S.I. LEAHY and G. W. THOMAS. Mycobacterium gastri peritonitis in a patient undergoing continuous ambulatory peritoneal dialysis. Aust NZ J Med 1986; 16
3. MT. LaRocco, JE. Mortenson. And A. Robinson. Mycobacterium fortuitum peritonitis in a patient undergoing chronic peritoneal dialysis. DIAGN MICROBIOL INFECT DIS. 1986; 4: 161-164
4. G.WOODS, G.HALL, AND MJ.SCHREIBER. Mycobacterium fortuitum Peritonitis Associated with Continuous Ambulatory Peritoneal Dialysis. JOURNAL OF CLINICAL MICROBIOLOGY. 1986; 23: 786-788
5. Mycobacterium chelonae peritonitis associated with continuous ambulatory peritoneal dialysis. TOBY L. MERLIN, M.D. AND ANTONIOS H. ACJP. 1989; 91: 718-720
6. F. Soriano, J. L. Rodriguez-Tudela, J. L. GOmez-Garc6s , M. Velo. Wo. Possibly Related Cases of Mycobacterium fortuitum Peritonitis Associated with Continuous Ambulatory Peritoneal Dialysis. Eur. J. Clin. Microbiol. Infect. Dis. 1989; 8: 895-897
7. JJ. Sennesael, VA. Maes, Denis Pierard, et al. Streptomycin pharmacokinetics in relapsing Mycobacterium xenopi peritonitis. Am J Nephrol. 1990;10: 422-425
8. RB. Dunmire, and JA. Breyer. Nontuberculous mycobacterial peritonitis during continuous ambulatory peritoneal dialysis case report and review of diagnostic and therapeutic strategies. American Journal of Kidney Diseases. 1991; 18: 126-130
9. M. Giladi, BE. Lee, OGW. Berlin, and CB. Panosian. Peritonitis caused by Mycobacterium kansasii in a patient undergoing continuous ambulatory peritoneal dialysis. American Journal of Kidney Diseases. 1992; 19: 597-599
10. HJ. KOLMOS, M. BRAHM and B. BRUUN. Peritonitis with Mycobacterium fortuitum in a patient on continuous ambulatory peritoneal dialysis. Scand J Infect Dis. 1992; 24: 801-803
11. Carl A. Perlino. Mycobacterium avium complex an unusual cause of peritonitis in patients undergoing continuous ambulatory peritoneal dialysis. Clinical Infectious Diseases 1993; 17: 1083-4
12. R White, K Abreo, R Flanagan, M Gadallah, et al.MD. Nontuberculous Mycobacterial Infections in Continuous Ambulatory Peritoneal Dialysis Patients. American Journal of Kidney Diseases. 1993; 22: 581-587
13. M. Perazella, T. Eisen, and E. Brown. Peritonitis associated with disseminated Mycobacterium avium complex in an acquired immunodeficiency syndrome patient on chronic ambulatory peritoneal dialysis. American Journal of Kidney Diseases. 1993; 21: 319-321
14. CW. Choi, DR Cha, YJ. Kwon, et al. Mycobacterium fortuitum peritonitis associated with continuous ambulatory peritoneal dialysis. The Korean J of Inter Med. 1993; 8: 25-27
15. Catheter-related Mycobacterium chelonei infection in a CAPD patient. Todd W.B. Gehr, Brian A.J. Walters. Peritoneal Dialysis International. 1994; 14: 278-288
16. L. Irizarry, J. Rupp. J. Smart, A. Nwosu, et al. Diagnostic difficulties with mycobacteria peritonitis in a CAPD patient. Peritoneal Dialysis International. 1996; 16: 427
17. C. Harro, GL. Braden, AB. Morris, et al. Failure to cure Mycobacterium gordonae peritonitis associated with continuous ambulatory peritoneal dialysis. Clinical Infectious Diseases 1997; 24: 955-7
18. Elahna Paul and Prasad Devarajan. Mycobacterium phlei peritonitis: a rare complication of chronic peritoneal dialysis. Pediatr Nephrol. 1998; 12: 67-68
19. KM SLAGLE, DL OBLACK. Mycobacterium abscessus peritonitis a case report. CLINICAL LABORATORY SCIENCE. 1998; 11: 206-208
20. G Vera, SQ Lew. Mycobacterium fortuitum peritonitis in two patients receiving continuous ambulatory peritoneal dialysis. Am J Nephrol 1999;19: 586-589
21. S. Kawamoto, K. Otani, Y. Kawaguchi, et al. Mycobacterium fortuitum peritonitis associated with CAPD diagnosis by a molecular biology technique. Perit Dial Int. 1999; 19(6): 592-3.
22. Hevia C, Bajo MA, Sánchez-Tomero JA, et al. Peritoneal catheter exit-site infections caused by rapidly growing atypical mycobacteria. Nephrol Dial Transplant. 2000;15(9):1458-1460.
23. S Osada, C Hamada, M Fukui, et al. A case of Mycobacterium fortuitum peritonitis associated with continuous ambulatory peritoneal dialysis (CAPD). Journal of Japanese Society for Dialysis Therapy. 2001; 34 (9): 1251-1255
24. JT. Youmbissi, QT. Malik, SK Ajit, et al. Non tuberculous mycobacterium peritonitis in continuous ambulatory peritoneal dialysis. J NEPHROL 2001; 14: 132-135
25. S. Tang, AW. Tang, XY Chang. Successful treatment of Mycobacterium fortuitum peritonitis without tenckhoff catheter removal in CAPD. Perit Dial In. 2003; 23(3): 304-5.
26. T Ando, M Ryuzaki, M Handa, et al. Mycobacterium fortuitum Peritonitis in a Patient Undergoing Continuous Ambulatory Peritoneal Dialysis. Hong Kong J Nephrol. 2003; 5: 101-104
27. Ferrara E, Lemire J, Grimm P, et al. Mycobacterial peritonitis in pediatric peritoneal dialysis patients. Pediatr Nephrol. 2004; 19: 114-117
28. Keenan N, Jeyaratnam D, Sheerin N.S. Mycobacterium simiae: a previously undescribed pathogen in peritoneal dialysis peritonitis. Am J Kidney Dis. 2005; 45: e75-78
29. Ellis EN, Schutze GE, Wheeler JG. Nontuberculous mycobacterial exit-site infection and abscess in a peritoneal dialysis patient. A case report and review of the literature. Pediatr Nephrol. 2005;20(7):1016-1018. doi:10.1007/s00467-005-1870-4
30. Gourtzelis N, Margassery S, Bastani B. Successful treatment of severe Mycobacterium fortuitum exit-site infection with preservation of the Tenckhoff catheter. Perit Dial Int. 2005;25(6):607-608.
31. Kameyama H, Mori Y, Kimura T, et al. A case report of Mycobacterium abscessus peritonitis in a peritoneal dialysis patient. Ther Apher Dial. 2007; 11: 449-51
32. Rho M, Bia F, Brewster UC. Nontuberculous mycobacterial peritonitis in peritoneal dialysis patients. Semin Dial. 2007; 20: 271-6
33. Tse K. C, Lui S. L, Cheng V. C, et al. A cluster of rapidly growing mycobacterial peritoneal dialysis catheter exit-site infections Am J Kidney Dis 2007
34. T. Hod, R. Kushnir, Y. Paitan and Z. Korzets. Mycobacterium fortuitum infection in continuous ambulatory peritoneal dialysis. Clinical Nephrology. 2008; 70: 546-553
35. Lee KF, Chen HH, Wu, CJ. Mycobacterium chelonae peritonitis in a patient on peritoneal dialysis. Ren Fail. 2008; 30: 335-8
36. EM Curry, M Yehia1, S Roberts. CAPD peritonitis caused by Mycobacterium rhodesiae. Perit Dial In. 2008; 28: 97-99
37. EL Falcone, A Alam and N Tangri. Mycobacterium avium complex associated peritonitis in a patient on continuous ambulatory peritoneal dialysis. Clinical Nephrology. 2008; 69: 387-390
38. Y Maeda, T Uno, A Yoshida, et al. Nontuberculous Mycobacterial Peritonitis in a Patient Undergoing Continuous Ambulatory Peritoneal Dialysis. J Rural Med 2009; 4(2): 75-79
39. Chung JW, Cha YJ, Oh DJ, et al. Disseminated Mycobacterium avium complex infection in a non-HIV-infected patient undergoing continuous ambulatory peritoneal dialysis. Korean J Lab Med. 2010; 30: 166-70
40. Patil R, Patil T, Schenfeld L, et al. Mycobacterium porcinum peritonitis in a patient on continuous ambulatory peritoneal dialysis. J Gen Intern Med. 2011; 26: 346-8
41. Chan WW, Murray MC, Tang P, et al. Mycobacterium heckeshornense peritonitis in a peritoneal dialysis patient: a case report and review of the literature. Clin Microbiol Infect. 2011; 17: 1262-1264
42. SH Jiang, S Senanayake, GS Talaulikar. Peritoneal dialysis-related peritonitis due to Mycobacterium smegmatis. Perit Dial Int. 2011; 31(2): 215-6.
43. Renaud CJ, Subramanian S, Tambyah PA, et al. The clinical course of rapidly growing nontuberculous mycobacterial peritoneal dialysis infections in Asians: A case series and literature review. Nephrology. 2011; 16: 174-9
44. MA Simbli, FA Niaz, JS. Al-Wakeel. Encapsulating Peritoneal Sclerosis in a Peritoneal Dialysis Patient Presenting with Complicated *Mycobacterium fortuitum* Peritonitis. Saudi J Kidney Dis Transpl. 2012; 23(3): 635-641
45. N Siddiqi, I Sheikh. Peritonitis Caused by *Mycobacterium abscesses* in Patients on Continuous Ambulatory Peritoneal Dialysis. Saudi J Kidney Dis Transpl. 2012; 23(2): 321-324
46. SH Jiang, DM Roberts, AH Dawson and M Jardine. Mycobacterium fortuitum as a cause of peritoneal dialysis-associated peritonitis: case report and review of the literature. BMC Nephrology. 2012; 13(35)
47. A Jo, Y Ishibashi, D Hirohama1, et al. Early Surgical Intervention May Prevent Peritonitis in Cases with Tenckhoff Catheter Infection by Nontuberculous Mycobacterium. Perit Dial Int. 2012; 32: 226-7
48. Lo MW, Mak SK, Wong YY. Atypical mycobacterial exit-site infection and peritonitis in peritoneal dialysis patients on prophylactic exit-site gentamicin cream. Perit Dial Int. 2013; 33: 267-72
49. Sangwan J, Lathwal S, Kumar S, et al. .Mycobacterium fortuitum Peritonitis in a Patient on Continuous Ambulatory Peritoneal Dialysis (CAPD): A Case Report. J Clin Diagn Res. 2013; 7: 2950-2951
50. Y Miyasato, M Adachi, Y Fujie, et al. Severe Mycobacterium fortuitum infection due to inappropriate exit-site care using mountain spring water in a patient on continuous ambulatory peritoneal dialysis（CAPD). Journal of Japanese Society for Dialysis Therapy. 2013; 6: 937-942
51. D Ranganathan, R Fassett, GT John. Mycobacterium fortuitum Peritonitis in a Patient Receiving Continuous Ambulatory Peritoneal Dialysis. Saudi J Kidney Dis Transpl. 2013; 24: 1003-1004
52. Jiang SH, Roberts DM, Clayton PA, et al. Non-tuberculous mycobacterial PD peritonitis in Australia. Int Urol Nephrol. 2013; 45: 1423-8
53. Tsai SF. Catheter related infection due to Mycobacterium abscessus in a patient under peritoneal dialysis. Ther Apher Dial. 2013;17(3):349-350.
54. Miyashita E, Yoshida H, Mori D, et al. Mycobacterium avium complex-associated peritonitis with CAPD after unrelated bone marrow transplantation. Pediatr Int. 2014; 56: e96-e98
55. Kunin M, Knecht A, Holtzman EJ. Mycobacterium chelonae peritonitis in peritoneal dialysis. Literature review. Eur J Clin Microbiol Infect Dis. 2014; 33: 1267-71
56. Hamade A, Pozdzik A, Denis O, et al. Mycobacterium fortuitum and Polymicrobial Peritoneal Dialysis-Related Peritonitis: A Case Report and Review of the Literature. Case Rep Nephrol. 2014; 2014: 323757
57. S Zewinger, CM Meier, D Fliser, et al. Mycobacterium fortuitum peritonitis in peritoneal dialysis and its effects on the peritoneum. Clinical Nephrology. 2014; 82: 341-346
58. Yamada T, Ushijima K, Uemura O. A hospital-acquired outbreak of catheter-related nontuberculous mycobacterial infection in children on peritoneal dialysis. CEN Case Rep. 2015; 4: 43-47
59. PL Lu, HT Kuo, MC Kuo, et al. Peritoneal Dialysis-Associated Peritonitis Caused by Mycobacterium abscessus. Perit Dial Int. 2015; 35: 366-9
60. Okado T, Iimori S, Nishida H, et al. Successful treatment of *Mycobacterium chelonae* peritoneal dialysis-related infection by a combination regimen including local thermal therapy. *Perit Dial Int*. 2015;35(1):114-116.
61. Martínez López AB, Álvarez Blanco O, Ruíz Serrano MJ, Morales San-José MD, Luque de Pablos A. *Mycobacterium fortuitum* as a cause of peritoneal dialysis catheter port infection. A clinical case and a review of the literature. *Nefrologia*. 2015;35(6):584-586.
62. Inagaki K, Mizutani M, Nagahara Y, et al. Successful Treatment of Peritoneal Dialysis-related Peritonitis due to Mycobacterium iranicum. 2016; 55: 1929-31
63. Choi HS, Bae EH, Ma SK, et al. Peritoneal Dialysis-Related Peritonitis Caused by Microbacterium paraoxydans. Jpn J Infect Dis. 2017; 70: 195-196
64. Fujikura H, Kasahara K, Ogawa Y, et al. Mycobacterium wolinskyi Peritonitis after Peritoneal Catheter Embedment Surgery. 2017; 56: 3097-3101
65. GCW Chan, MMY Mok, DLL Hung, et al. MYCOBACTERIUM CHLOROPHENOLICUM: AN UNCOMMON CAUSE OF PERITONITIS IN A PERITONEAL DIALYSIS PATIENT. Nephrology. 2017; 22: 498-499
66. Mooren Vhjf, Bleeker MWP, van Ingen J, et al. Disseminated Mycobacterium abscessus infection in a peritoneal dialysis patient. IDCases. 2017; 9: 6-7
67. Hibi A, Kasugai T, Kamiya K, et al. Peritoneal dialysis-associated catheter infection caused by *Mycobacterium abscessus* in an elderly patient who was successfully treated with catheter removal. *CEN Case Rep*. 2017;6(2):175-179.
68. Mooren VHJF, Bleeker MWP, van Ingen J, Hermans MHA, Wever PC. Disseminated *Mycobacterium abscessus* infection in a peritoneal dialysis patient. *IDCases*. 2017;9:6-7.
69. Koratala A, Chornyy V, Kazory A. Exit Site Infection in Peritoneal Dialysis; Need for Follow-Up on Cultures Despite Clinical Improvement. *Blood Purif*. 2017;44(1):66-67.
70. HH Chin, YH Chin, YL Yap, et al. Case Report: Two Cases of Mycobacterium Abscessus Peritonitis in Patients on Continuous Ambulatory Peritoneal Dialysis. J. of Nephrology Res. 2018; 4(1): 135-138
71. N Washidaa, H Itoh. The Role of Non-Tuberculous Mycobacteria in Peritoneal Dialysis-Related Infections: A Literature Review. Recent Advances in Dialysis Therapy in Japan. 2018; 196: 155-161
72. Yoshimura R, Kawanishi M, Fujii S, et al. Peritoneal dialysis-associated infection caused by Mycobacterium abscessus: a case report. BMC Nephrol. 2018; 19: 341
73. Ono E, Uchino E, Mori K, et al. Peritonitis due to Mycobacterium abscessus in peritoneal dialysis patients: case presentation and mini-review. Renal Replacement Therapy. 2018; 4: 52
74. H Inoue, N Washida, K Morimoto, et al. Non Tuberculous Mycobacterial Infections Related to Peritoneal Dialysis. Perit Dial Int. 2018; 38(2):147-149
75. Hibi A, Kasugai T, Kamiya K, et al. Exit Site Infection due to Mycobacterium chelonae in an Elderly Patient on Peritoneal Dialysis. *Case Rep Nephrol Dial*. 2018;8(1):1-9.
76. Shenoy A, El-Nahal W, Walker M, et al. Management of a Mycobacterium immunogenum infection of a peritoneal dialysis catheter site. *Infection*. 2018;46(6):875-880.
77. Marzuk SM, Rohit A, Nagarajan P, et al. An unusual case of unresolving tunnel infection in a patient on continuous ambulatory peritoneal dialysis. *Indian J Med Microbiol*. 2018;36(4):600-6
78. Jheeta AS, Rangaiah J, Clark J, et al. Mycobacterium abscessus - an uncommon, but important cause of peritoneal dialysis-associated peritonitis - case report and literature review. BMC Nephrol. 2020; 21: 491
79. Jung JH, Ahn SH. Peritoneal Dialysis Catheter-Related Infection due to Mycobacterium abscessus Confused with Rhodococcus. J Korean Med Sci. 2020; 35: e44
80. Seki M, Kamioka Y, Takano K, et al. Mycobacterium abscessus Associated Peritonitis with CAPD Successfully Treated Using a Linezolid and Tedizolid Containing Regimen Suggested Immunomodulatory Effects. 2020; 21: e924642
81. N Klomjit, A Chewcharat, M D'Uscio, et al. Mycobacterium septicum associated peritonitis: A case report. Peritoneal Dialysis International. 2020; 40(6): 600-602
82. Imam O, Al-Zubaidi K, Janahi M, et al. Peritoneal Dialysis-Associated Peritonitis Caused by Mycobacterium abscessus in Children-A Case Report. Open Forum Infect Dis. 2021; 8: ofaa579
83. Hamada S, Takata T, Kitaura T, et al. Peritoneal dialysis-associated peritonitis caused by Mycobacteroides massiliense: the first case and review of the literature. BMC Nephrol. 2021; 22: 90
84. Lu J, Jiang Z, Wang L, et al. Mycobacteria avium-related peritonitis in a patient undergoing peritoneal dialysis: case report and review of the literature. BMC Nephrol. 2021; 22: 345
85. Pinapala A, Koh LJ, Ng KH, et al. Clofazimine in Mycobacterium abscessus peritonitis: A pediatric case report. Perit Dial Int. 2021; 41: 104-109
86. Yokota S, Nishi K, Ishiwa S, et al. Mycobacterium avium complex peritonitis in a pediatric patient on peritoneal dialysis: A case report. Medicine. 2021; 100: e26321
87. Chang ZY, Tok PL, Teo BW, et al. Non-tuberculous mycobacteria infections in peritoneal dialysis: Lessons from a 16-year single-centre experience. Ann Acad Med Singap. 2021; 50: 724-728
88. Chamarthi G, Modi D, Andreoni K, Shukla AM. Simultaneous catheter removal and reinsertion, is it acceptable in *M. abscessus* exit site infection? *CEN Case Rep*. 2021;10(4):483-489.
89. K Masuda, Y Yamasaki. A successfuly treated case of PD‒related peritonitis caused by Mycobacterium wolinskyi. Journal of Japanese Society for Dialysis Therapy. 2022; 55: 249-253
90. Haubrich K, Mammen C, Sekirov I, et al. Mycobacterium fortuitum peritoneal dialysis-related peritonitis in a child: A case report and review of the literature. J Assoc Med Microbiol Infect Dis Can. 2022; 7: 125-130
91. Hayat A, Sakhrani B, Rubin M. Mycobacterium chelonae-related peritoneal dialysis peritonitis: a case report and its potential complications. Int Urol Nephrol. 2022; 54: 1769-1771
92. Nakano S, Miyazaki N, Michigami T, et al. A case of a preschool child with a successful kidney transplant following the long-term administration of antibiotics to treat peritoneal dialysis-related ESI/peritonitis by Mycobacterium abscessus. CEN Case Rep. 2022; 11: 408-411
93. Rouhani S, Adunuri N. Refractory Peritonitis and Small Bowel Ileus: A Case of Encapsulating Peritoneal Sclerosis Secondary to Mycobacterium abscessus Peritonitis. Eur J Case Rep Intern Med. 2022; 9: 003173
94. Ueda Y, Okamoto T, Sato Y, et al. Kidney transplantation after peritoneal dialysis-associated peritonitis and abdominal abscesses caused by Mycobacterium massiliense: lesson for the clinical nephrologist. J Nephrol. 2022; 35: 1907-1910
95. Bartolomeo K, Hassanein M, Vachharajani T. J. Management of peritoneal dialysis Mycobacterium abscessus exit-site infection: A case report and literature review. J Vasc Access 2022
96. Chen X, Zhu J, Liu Z, et al. Mixed infection of three nontuberculous mycobacteria species identified by metagenomic next-generation sequencing in a patient with peritoneal dialysis-associated peritonitis: a rare case report and literature review. BMC Nephrol. 2023; 24: 95
97. Takata K, Omae T, Hamano Y, et al. Peritoneal dialysis-associated infection caused by Mycobacterium abscessus in a pediatric patient on continuous peritoneal dialysis without switching to hemodialysis CEN Case Rep 2024
