# Supplementary figures and images for: Peritoneal Dialysis‐Related Mycobacterium fortuitum Exit‐Site/Tunnel Infection in a Pediatric Patient: A Case Report
Source: Clin Case Rep. 2026 Feb 12;14(2):e71975. doi: 10.1002/ccr3.71975 (PMC12901669; doi:10.1002/ccr3.71975)

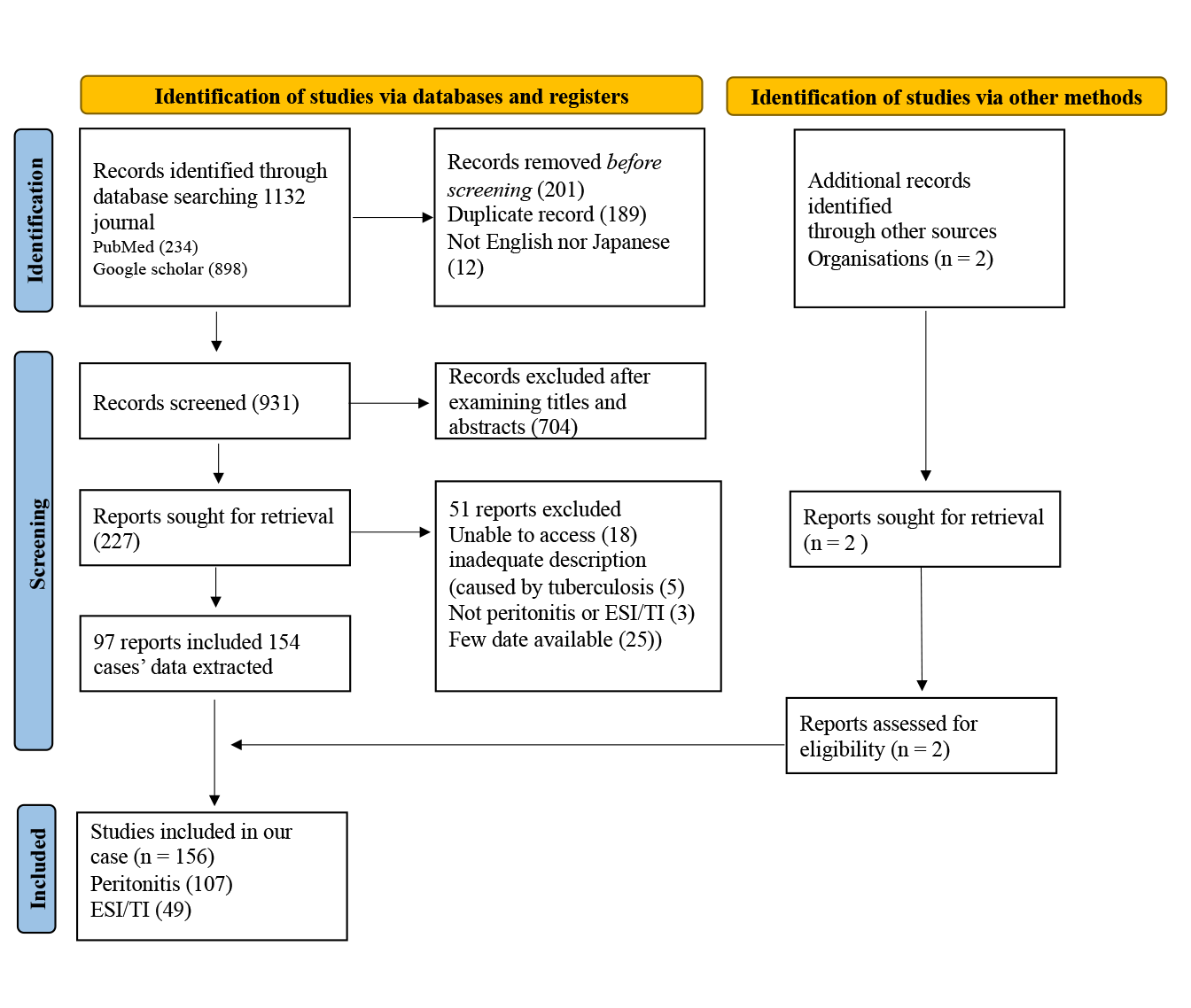

Supplement: Supplementary file 2 — Figure S1: Literature search strategy illustrating the databases searched, search terms, and selection process used to identify eligible studies for this review. [file CCR3-14-e71975-s002.tif]
